# Molecular origin of the Raman signal from *Aspergillus nidulans* conidia and observation of fluorescence vibrational structure at room temperature

**DOI:** 10.1038/s41598-020-62112-w

**Published:** 2020-03-25

**Authors:** Zehua Han, Benjamin D. Strycker, Blake Commer, Kai Wang, Brian D. Shaw, Marlan O. Scully, Alexei V. Sokolov

**Affiliations:** 1 Institute for Quantum Science and Engineering, Texas A&M University, College Station, Texas USA; 2 Baylor University, Waco, Texas USA; 3 Department of Plant Pathology and Microbiology, Texas A&M University, College Station, Texas USA

**Keywords:** Biophotonics, Biophysics

## Abstract

Successful approaches to identification and/or biological characterization of fungal specimens through Raman spectroscopy may require the determination of the molecular origin of the Raman response as well as its separation from the background fluorescence. The presence of fluorescence can interfere with Raman detection and is virtually impossible to avoid. Fluorescence leads to a multiplicity of problems: one is noise, while another is “fake” spectral structure that can easily be confused for spontaneous Raman peaks. One solution for these problems is Shifted Excitation Raman Difference Spectroscopy (SERDS), in which a tunable light source generates two spectra with different excitation frequencies in order to eliminate fluorescence from the measured signal. We combine a SERDS technique with genetic breeding of mutant populations and demonstrate that the Raman signal from *Aspergillus nidulans* conidia originates in pigment molecules within the cell wall. In addition, we observe unambiguous vibrational fine-structure in the fluorescence response at room temperature. We hypothesize that the vibrational fine-structure in the fluorescence results from the formation of flexible, long-lived molecular cages in the bio-polymer matrix of the cell wall that partially shield target molecules from the immediate environment and also constrain their degrees of freedom.

## Introduction

Fungi are ubiquitous both in natural and domestic environments, and their effects on human activity are numerous as well as widely varied. For example, fungi growing in outdoor environments may form mycorrhizal networks that form the basis for plant-fungal symbioses, allowing the exchange and/or transfer of nutrient and other important molecular resources^1–3^. Fungi have traditionally been used in the production of food products such as cheeses, soy sauce, miso, sonti, and tempeh. In addition, they are used in the industrial production of lactic, citric, acetic, and other acids, as well as antibiotics, vitamins, ethyl alcohol, amino acids, hormones, single-cell proteins, and fats^4^. In contrast, uncontrolled fungal growth can be costly to human health, agriculture, forestry, and livestock. For example, around 4.6 million people in the U.S. suffer a loss of ~$3.5 billion annually from mold-related asthma^5^. In total, mold exposure, infection, and damage lead to agricultural losses of millions of dollars to American agri-producers every year^6^. Consequently, public and economic health are harmed by detrimental mold and mycotoxins^7^.

In order to evaluate and/or prevent damage from mold growth, spore detection and species identification is paramount. We envision the development of a mobile Raman spectroscopy scheme for timely and on-site identification and/or biological characterization. Determination of the relevant experimental and physiological parameters, such as the molecular origin of the Raman signal, is therefore critical. Several groups have made progress in this endeavor. Ghosal *et al*. have used spontaneous Raman micro-spectroscopy to measure the spectra of seven mold species and to differentiate them from one another^8^. Similarly, Farazkhorasani measured and differentiated between the spontaneous Raman spectra of several strains of *Aspergillus nidulans*^9^. Surface-Enhanced Raman Spectroscopy (SERS) of the mold species *A. nidulans* has also been reported^9,10^. Raman spectroscopy has therefore been shown to be a robust technique for chemical identification and characterization^11,12^. However, Raman signals are not conspicuous when strong fluorescence emission is present, especially in biological specimens. Therefore, eliminating fluorescence is a challenge in Raman spectroscopy applications. Many techniques have been developed to overcome this challenge, such as time-gated Raman spectroscopy^13^ and coherent anti-Stokes Raman spectroscopy (CARS)^14^. However, these methods are costly, require sophisticated designs as well as layouts, and may induce additional problems, such as a non-resonant background in CARS. A simpler approach proposed by Shreve and coworkers is termed Shifted Excitation Raman Difference Spectroscopy (SERDS)^15–20^. This method relies on the fact that the Raman response is sensitive to slight changes in excitation frequency, while the fluorescence response is comparatively insensitive. Consequently, the difference between two measured spectra excited by slightly separated frequencies results in a derivative-like curve. The contribution from fluorescence is suppressed significantly in this difference spectrum, while the Raman contribution remains. Here, we use the same strategy to remove the fluorescence signal from conidia of *Aspergillus nidulans* (*A. nidulans*). We analyzed wild type *A. nidulans* conidia, which are naturally pigmented green, and compared them to two strains carrying pigment production mutations that do not fully form the green pigment and instead produce yellow or white spores. Additionally, conidia that either produced (*rodA*^+^) or lacked (Δ*rodA*) the RodA hydrophobin protein, a dominant feature of the surface of conidia, were also analyzed (see Appendix 2 in Supplementary Information).

One of our goals in comparing the obtained spectra was to determine the molecular origin of the Raman signal generated using 785 nm excitation radiation, thereby gaining valuable insight into how effective biosensing Raman schemes might be implemented in the future. Our selection of 785 nm excitation radiation in this work is motivated by several factors. Firstly, 785 nm radiation sources are relatively cheap and easy to implement. If Raman analysis of spores is ever to become a viable commercial technique, this must be taken into account. Secondly, 785 nm exhibits a reduced fluorescence response from biological samples. Thirdly, we wish to compare our results to those of previously published works which have also used 785 nm excitation radiation^8,9^. Taking into account both color and the existence/deletion of the RodA protein, we analyzed conidia from a total of six different strains. If the Raman signal originates from pigment molecules within the cell wall, the Raman spectra of different color phenotypes should exhibit marked differentiation. On the other hand, if the Raman signal originates from the surface of the cell wall, which is coated by hydrophobin proteins, the Raman spectra of ∆*rodA* mutants should significantly differ from their corresponding *rodA*^+^ counterparts. As outlined below, our results show that the Raman spectra varied with color phenotype rather than the presence or absence of RodA, indicating that the Raman signal originates not from hydrophobin proteins on the surface of the conidia, but rather from the pigments within the cell wall.

In addition, implementation of the SERDS technique has allowed us to observe unambiguous vibrational fine-structure in the fluorescence response at room temperature. This is highly unusual, since cryogenic temperatures are typically required to reduce the substantial inhomogeneous broadening that obscures the vibrational fine-scale fluorescence structure for molecules embedded in soft or condensed matter^21,22^. In what follows, we detail our results and hypothesize on a plausible mechanism.

## Methods and materials

### Sample preparation

The green wide-type (WT) strain (A4) was crossed with the white ∆*rodA* strain (FGSC A849)^23^ that carries mutants in both the *wA3* (white) and *yA2* (yellow) genes in order to produce both WT and ∆*rodA* with conidia of each of the three possible colors, as previously described^24^. These crosses were carried out on minimal medium (MM) with appropriate supplements. Each strain was evaluated based on previously published protocols^25^ for hydrophobicity to determine if it contained ∆*rodA*. Progeny were collected that displayed all six possible phenotypes: green, *rodA*^+^; green, ∆*rodA*; yellow, *rodA*^+^; yellow, ∆*rodA*; white, *rodA*^+^; and white, ∆*rodA*. To collect spores, each of the six strains was grown on an individual MM plate for seven days at 30 °C, then harvested with 1 mL sterile distilled water and a bent glass rod, for a final concentration of 1 × 10^6^ spores. Spore suspensions were kept at 4 °C while not in use, and adequately vortexed prior to preparing for experiments.

### SERDS experiments

**Figure 1:**
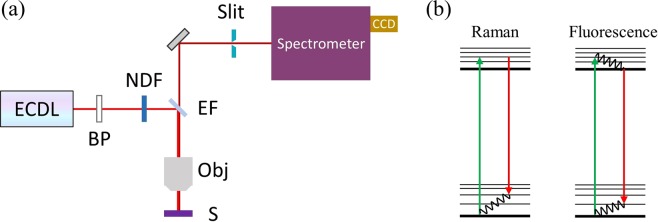
(**a**) Simplified schematic of SERDS experimental layout. ECDL: external cavity diode laser; BP: bandpass filter; NDF: neutral density filter; EF: edge filter; Obj: microscope objective; S: sample. (**b**) Diagrams of Raman scattering and fluorescence in a molecular system containing both electronic ground and excited states with multiple vibrational modes. Here, we assume that the excited electronic state contains a continuum of vibrational modes. The wavy lines indicate non-radiative decay.

The preparation of spore samples for subsequent Raman interrogation was completed as follows. First, ~6 μL of the spore suspension was pipetted onto the surface of an uncoated ultraviolet fused silica window (WG41010, Thorlabs). The water in the suspension was then allowed to evaporate over the course of several hours, leaving the spores as a deposit on the surface of the silica window. The spore sample on the silica window was then placed beneath a long-working-distance objective (HCX PL Fluotar, 100×, N.A. 0.75, Leica) in a commercial confocal Raman microscope (LabRAM HR Evolution, Horiba). The light source was a homemade external cavity diode laser (ECDL) operating at ~785 nm. Its characterization can be found in the Supplementary Table S1. A bandpass filter was inserted to clean the laser spectrum. Beam power was optimized to avoid burning the spore samples while maximizing the optical signal and was therefore set at ~0.87 mW. The Raman radiation was collected in the epi-direction (see Fig. 1(a)) and, after passing through an edge filter, propagated through the confocal slit (set to 200 μm), reflected off the spectrometer grating (150 gr/mm), and finally detected by an EMCCD (Synapse 354308, Horiba Jobin Yvon Inc.). Two spectra of a single spore excited by two different frequencies were recorded separately, the second immediately after the first. In order to increase the signal-to-noise ratio, each spectrum was taken as the average of 12 accumulations of 5-second acquisitions, for a total integration time of 60 seconds. Figure 1(b) demonstrates energy level diagrams of processes of Raman scattering and fluorescence in a molecular system.

## Results and discussion

**Figure 2:**
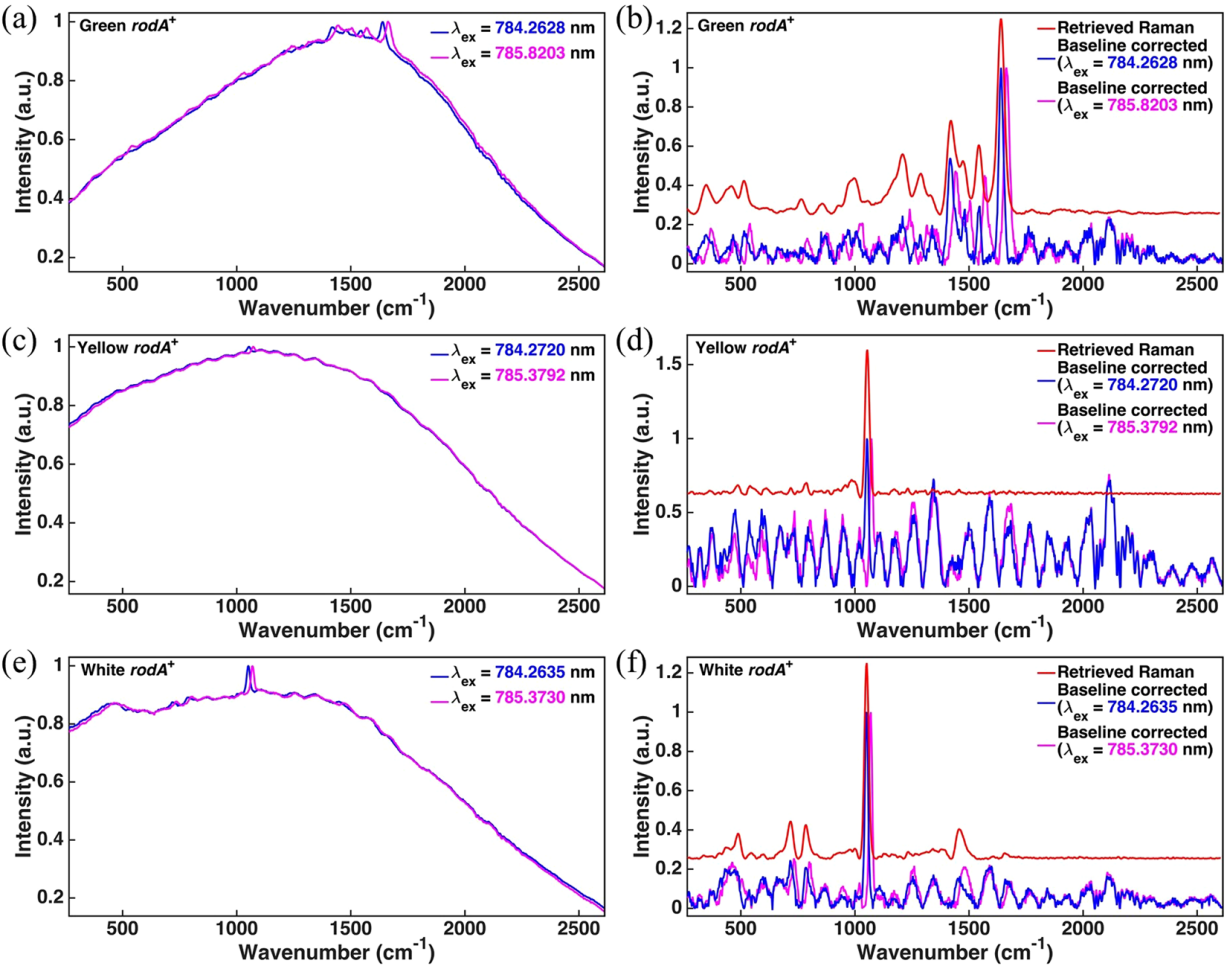
Spectra of color phenotypes and the corresponding SERDS spectra. The top (**a**,**b**), middle (**c**,**d**), and bottom (**e**,**f**) rows correspond to green, yellow, and white color phenotypes, respectively. The left column (**a**,**c**,**e**) corresponds to raw measured spectra taken with two slightly different laser excitation wavelengths. The right column (**b**,**d**,**f**) shows the corresponding reconstructed Raman spectrum (top red curve in each frame) retrieved using the SERDS technique compared to spectra generated from a conventional Asymmetric Least Squares (AsLS) algorithm (bottom curves in each frame). For the baseline-corrected spectra, only those features in each spectrum that correspond to a Raman response are sensitive to slight changes in laser excitation frequency.

For each of the three possible color phenotypes (green, yellow and white, respectively) of *A. nidulans* conidia, we analyzed 100 individual spores of *rodA*^+^ and 100 individual spores of Δ*rodA*. Within a given color phenotype, the Raman spectrum of the *rodA*^+^ is identical to that of its corresponding Δ*rodA* strain (see Supplementary Figs. S6(d), S7(d), and S8(d)). The remainder of this work therefore focuses only on strains that are *rodA*^+^. Raman data for each of three possible color phenotypes are shown in Fig. 2. It can be inferred that the genotype of all strains discussed from this point on are *rodA*^+^ strains unless otherwise specified. We point out that the measured raw spectra shown in Fig. 2 contain many fringe-like minor peaks that have the same spectral width as Raman peaks but which, in contrast to a genuine Raman response, are insensitive to slight changes in excitation frequency. We have taken care to establish that these minor peaks do not originate from any systematic instrumental response (see Appendix 4 in Supplementary Information). On the contrary, they are indicative of the light-matter interaction between the laser excitation and the molecules within the conidia. Because these minor peaks are insensitive to slight changes in excitation frequency, we conclude that they originate from the fluorescence response. Also, because they are superimposed over a much broader fluorescence background, we describe them using the term “fine-scale”. We demonstrate that a SERDS technique can separate these fine-scale fluorescence features from the Raman spectrum, while a conventional Asymmetric Least Squares (AsLS) background subtraction algorithm cannot^26^.

Figure 2(a) shows the normalized raw data of a single green spore excited by two slightly different frequencies. Each normalized raw spectrum was obtained by dividing the whole signal integrated from 250 cm^−1^ to 2600 cm^−1^ and then rescaling between 0 and 1, unless otherwise mentioned. We note that the frequency shift $\triangle v$ is not a bounded parameter in the SERDS method. Many studies have shown that the optimal value of $\triangle v$ should be comparable to the full-width-at-half-maximum (FWHM) of the major peaks^16,18^. Here, we set the frequency shift of the two excitation wavelengths (784.2628 nm and 785.8203 nm, respectively) to be ~25.2 cm^−1^, which is close to the bandwidth of the most prominent peak at ~1641 cm^−1^. Green spores exhibit strong fluorescence with four main peaks and a few minor peaks. Farazkhorasani^9^ has identified the main peak at ~1641 cm^−1^ as belonging to the Amide I C = C stretching mode. It should be noted that the molecular structure of the green pigment in *A. nidulans* is still unknown^27^. However, based upon its Raman spectrum, it should be expected that it contains an Amide group. The fluorescence background in the two measurements shown is almost identical, while several of the peaks are shifted. The reconstructed Raman spectrum shown in Fig. 2(b) (red curve) is generated through integration of the difference spectrum between two curves in Fig. 2(a) (see Appendix 1 and 5 in Supplementary Information). Conventional background-subtracted spectra (bottom curves in Fig. 2(b)) are also generated using the AsLS algorithm developed by Eilers and Boelens^26^ for comparison. The main difference between the two techniques is located in the region beyond 1700 cm^−1^. The restored Raman spectrum generated with the SERDS method shows no bands in this range. On the other hand, baseline-corrected spectra using the AsLS method display distinct and reproducible features in this region. However, these features remain unchanged as excitation frequency shifts, proving that they do not belong to Raman bands and are artifacts of fluorescence. Similar results are exhibited in the other 99 examined spores.

Figure 2(c) shows the normalized average raw data of 100 yellow spores with two different excitation wavelengths at ~785 nm. Here, the averaged raw data are used in order to improve the signal-to-noise ratio, which is otherwise low for a single spore. We set the shift of the two excitation frequencies to be ~18.0 cm^−1^, which is close to the bandwidth of the peak at ~1055 cm^−1^. Yellow spores have a strong fluorescence background and a small peak at ~1055 cm^−1^ that most likely corresponds to a C-O stretching mode^9^. The fluorescence signals in the two measurements are almost identical, while the peak exhibits a shift. The SERDS spectrum is also presented in the Supplementary Appendix 6. The reconstructed Raman spectrum in Fig. 2(d) (red curve) is generated by integrating the SERDS spectrum resultant from the spectra in Fig. 2(c). Also shown in Fig. 2(d) are conventional background-subtracted spectra (bottom curves) generated from the same data for comparison. As can be clearly seen, the two techniques manifest distinct differences. The restored Raman spectrum using the SERDS method shows only one noticeable band in the whole range. In contrast, the baseline-corrected spectra using the AsLS method present many features that remain unchanged as excitation frequency shifts, proving once again that they do not belong to Raman bands but in fact result from a fine-scale fluorescence response.

Figure 2(e) shows the normalized average raw data of 100 white spores. Once again, the averaged raw data are used in order to improve the signal-to-noise ratio, which is otherwise low for a single spore. Two different excitation wavelengths at ~785 nm were used, with a corresponding frequency shift of ~18.0 cm^−1^. White spores exhibit strong fluorescence along with a small peak at ~1051 cm^−1^. The SERDS spectrum is also presented in the Supplementary Appendix 7. The reconstructed Raman spectrum shown in Fig. 2(f) (red curve) is generated from the integration of the SERDS spectrum resultant from the data in Fig. 2(e). Also shown in Fig. 2(f) are conventional background-subtracted spectra (bottom curves) generated from the same data for comparison. The restored Raman spectrum using the SERDS method shows one noticeable band at ~1051 cm^−1^ and two small bands at ~717 cm^−1^ and 785 cm^−1^, respectively. The peak at ~1051 most likely corresponds to a C-O stretching mode^9^. The small bands at ~717 cm^−1^ and 785 cm^−1^ may tentatively be identified as belonging to phospholipid C-N stretching and O-P-O stretching, respectively^28^. As in the cases of green and yellow conidia, the baseline-corrected spectra of white conidia exhibit many features that remain unchanged as the excitation frequency shifts, proving that they are in fact fine-scale features of the fluorescence response.

Taking into account all of the above, it can be concluded that the measured Raman spectrum originates not from the hydrophobin proteins on the surface of the conidia but rather from the pigments within the cell wall, which result in each color phenotype. Our results are indicative of the biosynthetic pathway that produces each of the pigments. The white (*wA3*) and yellow (*yA2*) color phenotypes exist as mutations of the wild green phenotype and correspond to various stages of the pigment-producing process. An enzyme (WA) converts the white pigment molecule into the yellow pigment molecule, while an additional enzyme (YA) converts the yellow pigment molecule into the green pigment molecule, whose chemical structure is still unknown^27^. The green phenotype is expressed if the entire biosynthetic pathway is completed, while the white and yellow phenotypes are arrested at their corresponding stages. Our results show that the Raman spectrum of the white phenotype is noticeably different from that of the yellow phenotype, confirming a differing molecular composition and/or structure. Likewise, the Raman spectrum of the green phenotype is drastically different from both the white and the yellow phenotypes, again confirming a unique molecular composition and/or structure.

**Figure 3:**
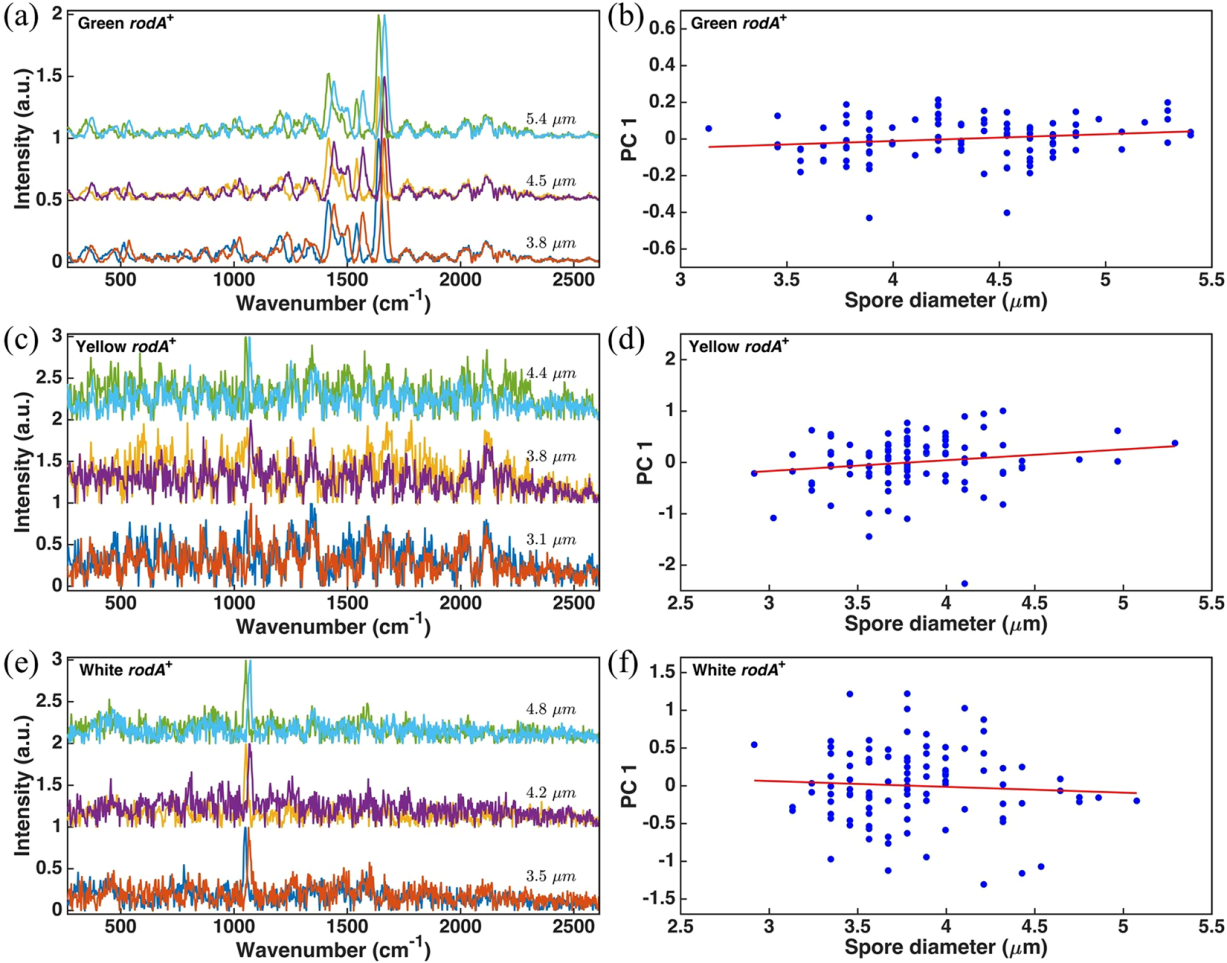
The fine-scale fluorescence spectra of three color phenotypes with different spore diameters and the PC score of the first principal component of the fine-scale fluorescence of 100 conidia versus their diameters. The numbers above the curves in (**a**,**c**,**e**) are the measured diameters of the corresponding spores. In order to increase the signal-to-noise ratio, spectra in (**c**,**e**) are averaged over the spores whose diameters are within ± 0.1 µm of the listed diameter.

The existence of fine-scale fluorescence features in the measured raw spectra that are otherwise indistinguishable from Raman peaks has no well-documented explanation. It can be seen from Fig. 2 that the spectral shape of the fine-scale fluorescence appears to be roughly periodic in nature. We have taken care to eliminate the possibility that these features might result from an instrumental or systematic anomaly in our equipment. One might suspect that the periodic structure results from spectral interference due to the changes in refractive index at the boundaries of the spore, but in fact the periodic spectral structure is almost identical in spores from the same population sample regardless of spore size, as shown in Fig. 3.

In order to determine if the size of the spores affects the fine-scale fluorescence, the spectra of each color phenotype obtained with slightly shifted excitation wavelengths have been plotted in Fig. 3(a,c,e). The spectra of each spore were obtained by subtracting its corresponding baseline using AsLS and then plotted according to different spore diameters. The diameter of each spore was measured according to the largest horizontal dimension. In addition, in Fig. 3(c,e), an average was taken over the spectra obtained from spores with diameters within ± 0.1 µm of the listed diameter in order to increase signal-to-noise ratio. The curves in Fig. 3(a,c,e), respectively, demonstrate that there is no distinct difference in the fine-scale fluorescence of each color phenotype as the spore diameter varies. Principle component analysis (PCA) of 100 spores of each color phenotype was also conducted in order to quantify the relationship of the fine-scale fluorescence to the spore diameter. Here, the spectrum corresponding to the lower excitation wavelength of each spore was used to construct a data matrix for PCA analysis with a Matlab software package (*pca* function)^29^. For convenience, we consider the fine-scale fluorescence located in the range from 1730 to 2600 cm^−1^. Figure 3(b,d,f) plot the PC score of the first principal component versus its corresponding spore diameter for different color phenotypes. The dependence of the fine-scale fluorescence on spore diameter is very weak. For example, the variation of fine-scale fluorescence in spore diameter has a Pearson correlation coefficient of *ρ* = 0.1667 with *R*^2^ = 0.0278 for green conidia and that of *ρ* = 0.1694 with *R*^2^ = 0.0287 for yellow conidia. In contrast, the fine-scale fluorescence of white conidia is even less dependent on spore diameter with *ρ* = −0.0635 and *R*^2^ = 0.0050. It is obvious that there is no correlation between fine-scale fluorescence and spore diameter in any color phenotype.

In addition, it is known that 785 nm radiation is efficiently scattered from the carbohydrates in cell walls, giving rise to the well-known red-edge effect of vegetation spectroscopy. Consequently, there are no surface waves to generate a corresponding spectral interference by propagating around the circumference of the spore. Finally, the spore diameter itself, 3 to 5 μm, is of the same order of magnitude as the wavelength of light used to interrogate it. It is simply impossible to account for the pathlength difference required to form the number of periodicities observed in the 1700 to 2500 cm^−1^ range of Fig. 2. It must be concluded, therefore, that these fine-scale spectral features do in fact result from a fundamental energy structure in the fluorescence response due to fluorescent radiative decay from the electronic excited state to various vibrational energy levels in the electronic ground state.

This conclusion, however, challenges current understandings of molecular dynamics in soft and condensed matter systems. It is widely acknowledged that molecules embedded in liquid or solid environments at room temperature do not exhibit vibrational fine-structure in their fluorescence spectra due to the substantial inhomogeneous broadening that obscures these fine-scale features^21,22^. The fact that we clearly observe vibrational fine-structure in the fluorescence response from molecules embedded in the cell wall of spores requires an unconventional explanation.

One possible explanation for the observed vibrational fine-structure in the fluorescence may be the formation of long-lived molecular cages in the bio-polymer matrix of the cell wall, as explored by Cicogna *et al*.^30^ and Prampolini *et al*.^31^. In these works, it was observed that chromophores embedded in polymer matrices exhibited vibrational fine-structure in their fluorescence spectra. Subsequently, through detailed discrete Fourier transform calculations, it was found that fluorescent molecules enclosed within a flexible cage-like structure formed by the polymer bundle were both partially shielded from direct interaction with the immediate environment and also slightly constrained in vibrational degrees of freedom, thus reducing the amount of inhomogeneous broadening that would otherwise obscure the vibrational structure in the fluorescence spectrum^30^. It is probable that these same physical conditions are prevalent within the cell wall of *A. nidulans*, which is largely composed of an extensively cross-linked glucan, chitin, and glycoprotein bio-polymer^32^.

If so, our unambiguous measurements of vibrational fine-structure in the fluorescence spectra of molecules embedded in conidia cell walls may provide the first observations of a possible mechanism for the allowance of long-lived quantum coherences in biological systems. Bio-polymers are ubiquitous in nature. It is not impossible that long-lived molecular cages play an important role in avian, insect, and plant magnetoreception^33,34^, for which coherence times as long as 10 μs may be required^35^.

## Conclusion

We have used the SERDS technique to successfully retrieve the Raman spectra of conidia of three possible color phenotypes of *rodA*^+^ strains in *A. nidulans*, as well as their corresponding ∆*rodA* strains. Our data indicate that, with an excitation wavelength of 785 nm, the Raman spectra of *A. nidulans* green *rodA*^+^ conidia originate in the pigment molecules within the cell wall. While the 785 nm wavelength used to generate Raman spectra in this work cannot distinguish *rodA*^+^ conidia from their ∆*rodA* counterparts, it may be possible to differentiate between *rodA*^+^ and ∆*rodA* conidia of the same color using a laser excitation frequency in the ultraviolet, since Raman scattering from proteins is resonantly enhanced in this regime.

In our experiments the SERDS technique requires a total acquisition time of 120 s compared to a 1 s dwell time in CARS experiments^14^, limiting its applications in fast imaging of biological samples such as observations of molecular dynamics. In addition, the quality of the spectral reconstruction depends on the amount of noise in the raw data. However, averaging over many samples as we have done here can increase the signal-to-noise level.

It should be noted that our measurements demonstrate SERDS has a distinct advantage over conventional baseline correction methods and that it is much more reliable and accurate. Baseline correction is unable to exclude fine-scale features in the measured spectrum that result from the fluorescence response, thus making the technique suspect when analyzing biological samples of the kind described in this work. In particular, software packages such as those used by both Ghosal *et al*.^8^ and Farazkhorasani^9^ may include erroneous fluorescence features in the retrieved spectrum. SERDS, on the other hand, can eliminate these fluorescence features from the measured spectrum because, in contrast to the desired Raman response, even the fine-scale fluorescence is insensitive to slight changes in the excitation frequency.

Finally, through implementation of the SERDS technique, we have unambiguously measured vibrational fine-structure in the fluorescence response of molecules embedded in conidia cell walls at room temperature. We hypothesize that this unusual result may be due to the formation of flexible, long-lived molecular cages within the bio-polymer of the cell wall, and that this effect may possibly play an important role in biological processes for which long-lived quantum coherence is required.

## Supplementary information

Supplementary Information.

## Data Availability

The datasets generated during and/or analyzed during the current study are available from the corresponding authors on request.
