# Supplementary material for: Molecular origin of the Raman signal from Aspergillus nidulans conidia and observation of fluorescence vibrational structure at room temperature
Source: Sci Rep. 2020 Mar 25;10:5428. doi: 10.1038/s41598-020-62112-w (PMC7096407; doi:10.1038/s41598-020-62112-w)
Supplement: Supplementary file 1 — Supplementary Information. [file 41598_2020_62112_MOESM1_ESM.pdf]

Supplementary Information

Molecular origin of the Raman signal from *Aspergillus nidulans* conidia and observation of fluorescence vibrational structure at room temperature

Zehua Han, [1] Benjamin D. Strycker, [1,2] Blake Commer,[3] Kai Wang,[1] Brian D. Shaw,[3]
Marlan O. Scully,[1,2] and Alexei V. Sokolov[1,2]
[1]Institute for Quantum Science and Engineering, Texas A&M University, College Station, Texas, USA
[2]Baylor University, Waco, Texas, USA
[3]Department of Plant Pathology and Microbiology, Texas A&M University, College Station, Texas, USA

Appendix 1: SERDS theory

The total signal S recorded by a spectrometer can be expressed with respect to the excitation frequency v_i as

$$S(v,\, v_i) = R(v,\, v_i) + L(v,\, v_i) + B(v)\,, \tag{1}$$

where R, L, and B represent the Raman signal, fluorescence signal, and background signal, respectively.

For two slightly different excitations v_1 and $v_2 = v_1 + \Delta v$, we have

$$S(v,\, v_1) = R(v,\, v_1) + L(v,\, v_1) + B(v)\,, \tag{2}$$
$$S(v,\, v_2) = R(v,\, v_2) + L(v,\, v_2) + B(v)\,. \tag{3}$$

The difference spectrum ΔS can then be derived:

$$\Delta S \equiv S(v,\, v_2) - S(v,\, v_1) = [R(v,\, v_2) - R(v,\, v_1)] + [L(v,\, v_2) - L(v,\, v_1)]\,. \tag{4}$$

Here, we assume that (1) laser power does not change when frequency varies slightly; (2) Raman and fluorescence signals are linearly dependent on laser power; (3) the Raman spectrum shifts the same amount as the laser frequency, while the fluorescence spectrum remains stationary. Mathematically, these assumptions imply:

$$R(v, v_2\,) = R(v, v_1 + \Delta v) = R(v + \Delta v, v_1)\,, \tag{5}$$
$$L(v,\, v_2) = \; L(v, v_1)\,. \tag{6}$$

Equation (4) then takes the form:

$$\Delta S = R(v, v_2) - R(v, v_1) = \; R(v + \Delta v, v_1) - R(v, v_1) \approx R'(v, v_1)\, \Delta v\,, \tag{7}$$

where the prime denotes the derivative with respect to frequency v. In Eq. (7), higher-order terms of Δv are neglected, since Δv itself is assumed to be small. The Raman signal can therefore be reconstructed as

$$R(v, v_1) \approx \int R'(v, v_1) dv = \frac{1}{\Delta v} \int \Delta S\; dv\,. \tag{8}$$

We note that the above method is equivalent to “Difference-Integration-δ_Deconvolution” [1].

Appendix 2: RodA hydrophobin

The gene *rodA* of *A. nidulans* encodes a small, moderately hydrophobic polypeptide [3]. Classified as a hydrophobin protein, the polypeptide is involved in the formation of filamentous rodlets on the surface of the *A. nidulans* conidia [3-6]. The rodlet nanostructure is significantly hydrophobic and allows for more efficient dispersal of the conidium throughout the local environment [3, 4]. The deletion of the *rodA* gene abolishes the production of the corresponding polypeptide, which in turn inhibits the formation of the hydrophobic protein nanostructure on the surface of each conidium, and results in a comparatively smooth surface that is much less hydrophobic [3-6]. *A. nidulans* strains containing this deletion are termed Δ*rodA*, while their unmodified counterparts (which are otherwise considered isogenic with the mutants) are termed *rodA*$^{+}$.

Appendix 3: External cavity diode laser (ECDL)

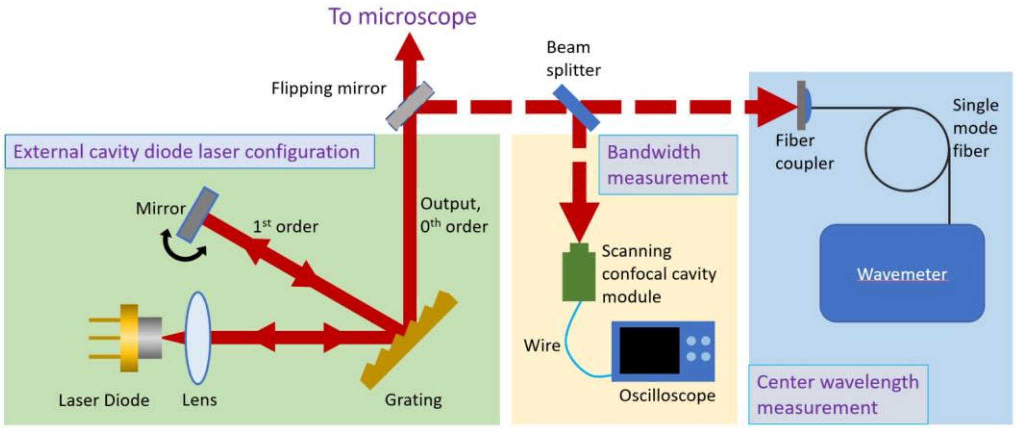

Figure S1. Schemes of ECDL, wavelength and bandwidth measurements.

There are two commonly used configurations in ECDL applications: Littrow configuration and Littman-Metcalf configuration [7, 8]. Taking into account both output efficiency and the requirement of a fixed output beam propagation-direction for varying wavelength, we abandoned the former option and chose the latter geometry. Our homemade ECDL consisted of a laser diode (L785P090, Thorlabs) operating at ~785 nm, a collimating lens, a diffraction grating, and an end mirror (see left panel in Figure S1). The zero-order diffracted beam served as the output, while the first-order diffracted beam was reflected back into the diode as feedback. The output wavelength could be continuously tuned by adjusting the angle of the end mirror on the first-order diffracted beam. The linewidth and central wavelength were also diagnosed. Laser linewidth was monitored by an oscilloscope, which was connected to a scanning confocal cavity module including a scanning Fabry-Perot interferometer (SCC-2500, VitaWave) and a scanning cavity driver (CFPD200, VitaWare) (Figure S1, middle panel). The central wavelength was measured by a wavemeter (WA-1500, Burleigh Instruments Inc.) (Figure S1, right panel).

We selected four wavelengths with a spacing of ~5 cm^{-1}. The frequency stability and spectral linewidth measurements were conducted simultaneously once a desired wavelength was achieved. In order to ensure the stability of the homemade laser during spore measurements, the characterizations for each wavelength lasted at least one minute. Table S1 summarizes the results of the frequency stability and spectral linewidth analyses of our homemade ECDL. The laser had a linewidth less than 150 MHz, and its frequency stability was within 150 MHz. It should be noted that Raman spectroscopy requires a light source with frequency stability better than 1 cm^{-1} (or 30 GHz) as well as a linewidth narrower than 30 GHz [7]. Therefore, our homemade ECDL was sufficient for Raman applications. We also investigated the long-term stability of the laser (see the last two rows in Table S1). The laser was inspected after 7 and 20 hours, respectively, of continuous operation. Although the central wavelength drifted slightly (which otherwise might result in serious consequences, such as spurious Raman shift and line broadening [7]), this small change (0.03 cm^{-1}) could be ignored in short one-minute detection acquisitions. These results confirmed that the tunable laser was stable and thus suitable for shifted excitation Raman difference spectroscopy (SERDS).

Wavelength (nm)		Frequency Fluctuation in 1 minute (MHz)	Spectral linewidth (MHz)	Frequency shift with respective to λ_1 (cm^{-1})
λ_1	785.2181	112	~ 150	0
λ_2	785.5506	68	~ 150	5.39
λ_3	785.8878	139	~ 150	10.85
λ_4	786.1166	155	~ 150	14.56
	786.1162*	91	~ 150	14.55
	786.1149**	144	~ 150	14.53

Table S1. Characterization of the homemade ECDL. Stability and linewidth of four different wavelengths as examples were inspected. * denotes the diagnosis was made after the ECDL had been running for 7 hours. ** represents inspections taken after 20 hours.

Appendix 4: Substrate

In order to determine the physical origin of the minor peaks in the measured raw spectra that were insensitive to slight changes in the excitation frequency, we compared magnified spectrum amplitude of substrate and spectrum amplitude of a single green *rodA*$^{+}$ spore (shown in Figure S2(a)). A similar baseline subtraction using the AsLS method was also used to determine if there was any fine-scale structure beyond 1700 cm^{-1} (shown in Figure S2(b)). The substrate did not exhibit the same behavior evident in spores, even though its signal was magnified by two orders of magnitude, implying that the fine-scale features in the raw spectra of sample conidia do not

originate from any systematic instrumental response but rather from the light-matter interaction between the laser excitation and the molecules within the conidium itself.

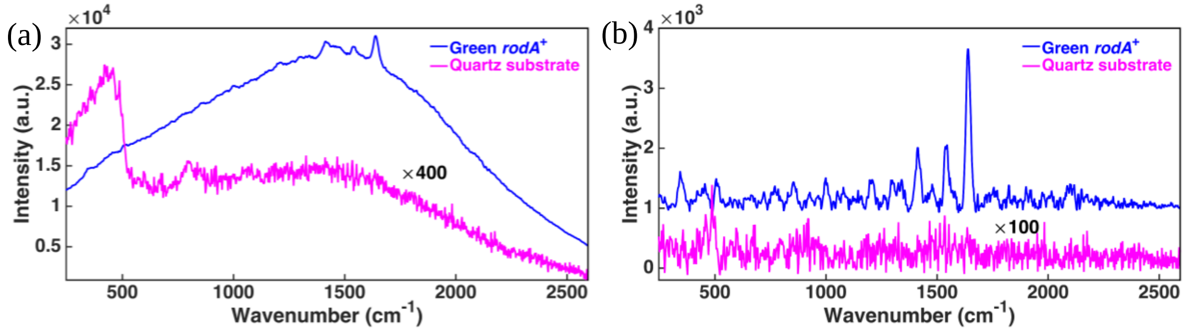

Figure S2. Comparison of green *rodA*⁺ spectrum and quartz spectrum. (a) Raw data of green *rodA*⁺ and magnified quartz signal. (b) Comparison of baseline-corrected green *rodA*⁺ spectrum and magnified background-free quartz spectrum.

Appendix 5: Green *rodA*⁺ conidia

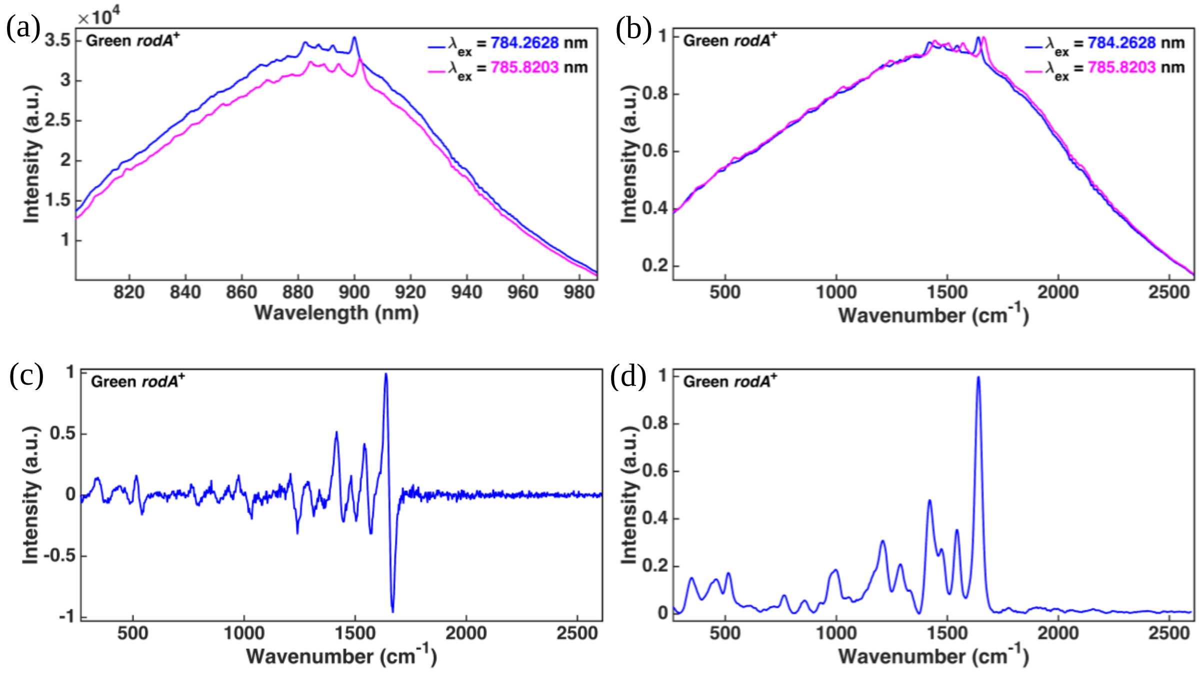

Figure S3. Results of a single green *rodA*⁺ conidium. (a) Raw spectra. (b) Normalized raw data corresponding to (a). (c) SERDS spectrum obtained by subtraction of two spectra in (b). (d) Retrieved spectrum from (c).

Figure S3(a) and (b) show the measured raw data and corresponding normalized spectra of a green *rodA*⁺ spore, respectively. Figure S3(c) is the SERDS spectrum with fluorescence removal. It was obtained by subtracting one spectrum from the other in Figure S3(b). The corresponding reconstructed spectrum is displayed in Figure S3(d). The relatively featureless region beyond 1700 cm^{-1} implies that there are no Raman bands in this range.

Appendix 6: Yellow *rodA*⁺ conidia

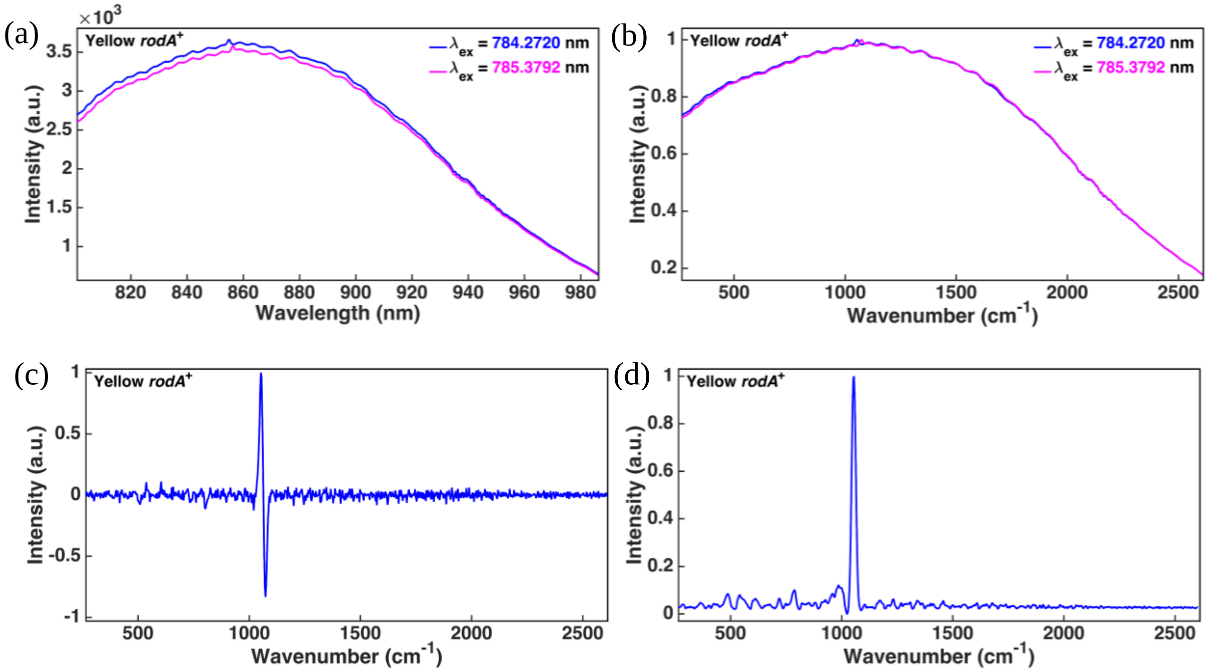

Figure S4. The average results of 100 yellow *rodA*⁺ conidia. (a) The average of raw data of 100 spores. (b) Normalized raw data corresponding to (a). (c) SERDS spectrum obtained by subtraction of two spectra in (b). (d) Retrieved spectrum from (c).

Figure S4(a) and (b) displays the average raw data and normalized raw data of 100 yellow rodA+ spores, respectively. Figure S4(c) shows the SERDS spectrum of yellow *rodA*⁺ spores free from fluorescence signals. Figure S4(d) is the retrieved Raman spectrum obtained by integrating the curve in Figure S4(a). The relatively featureless region beyond 1700 cm^{-1} implies that there are no Raman bands in this range, which is also consistent with the SERDS spectrum.

Appendix 7: White *rodA*$^+$ conidia

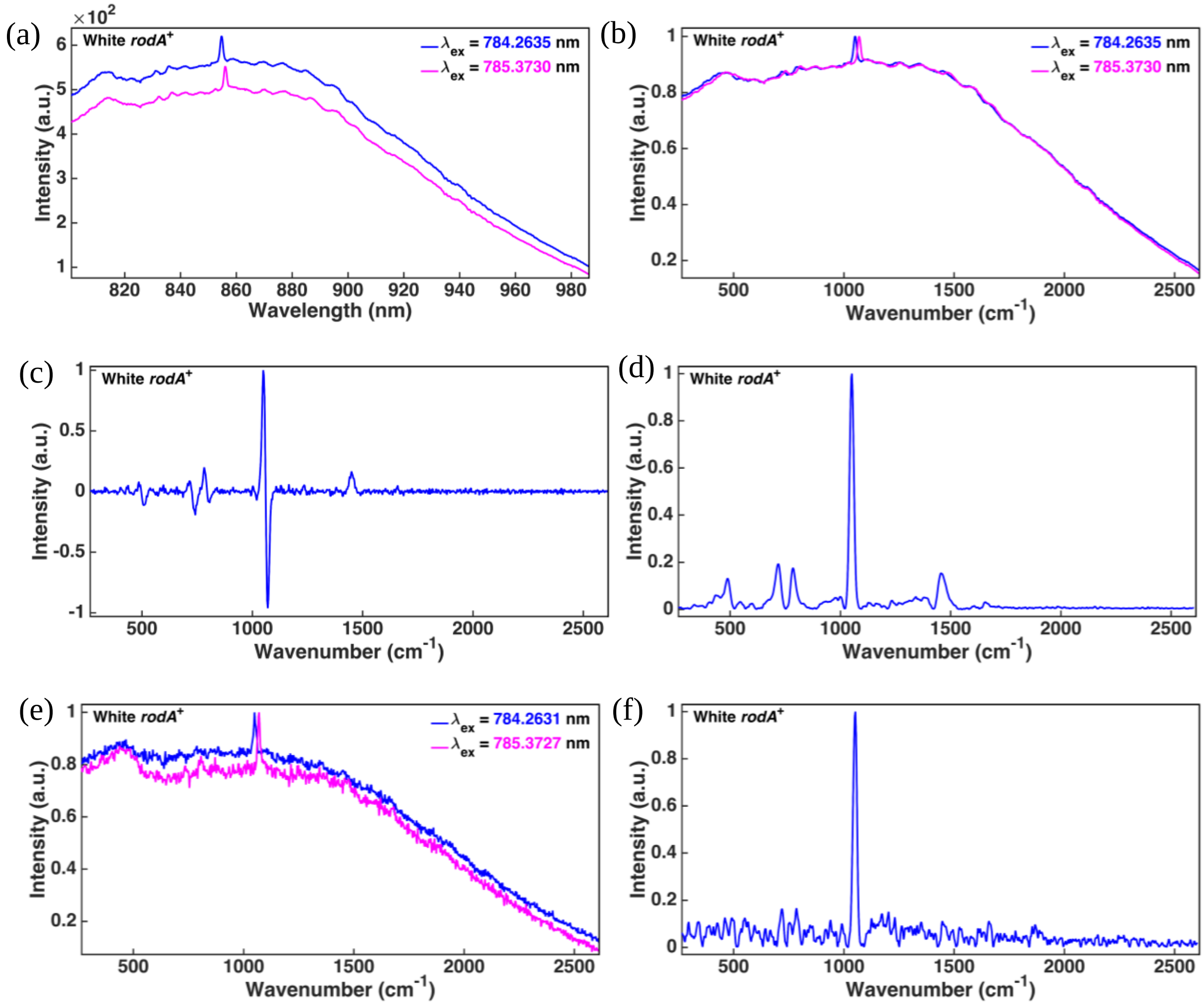

Figure S5. The average results of 100 white *rodA*$^+$ conidia (a), (b), (c), (d) and the results of a single spore (e) and (f) for comparison.

The results of 100 white *rodA*$^+$ spores are shown in Figure S5(a), (b), (c) and (d). For comparison, the results of a single conidium are also graphed in Figure S5(e) and (f). When Raman signals at ~717 cm^{-1} and 785 cm^{-1} are comparable with noise level as plotted in Figure S5(e), the reconstructed Raman spectrum cannot resolve these bands.

Appendix 8: Green Δ*rodA* conidia

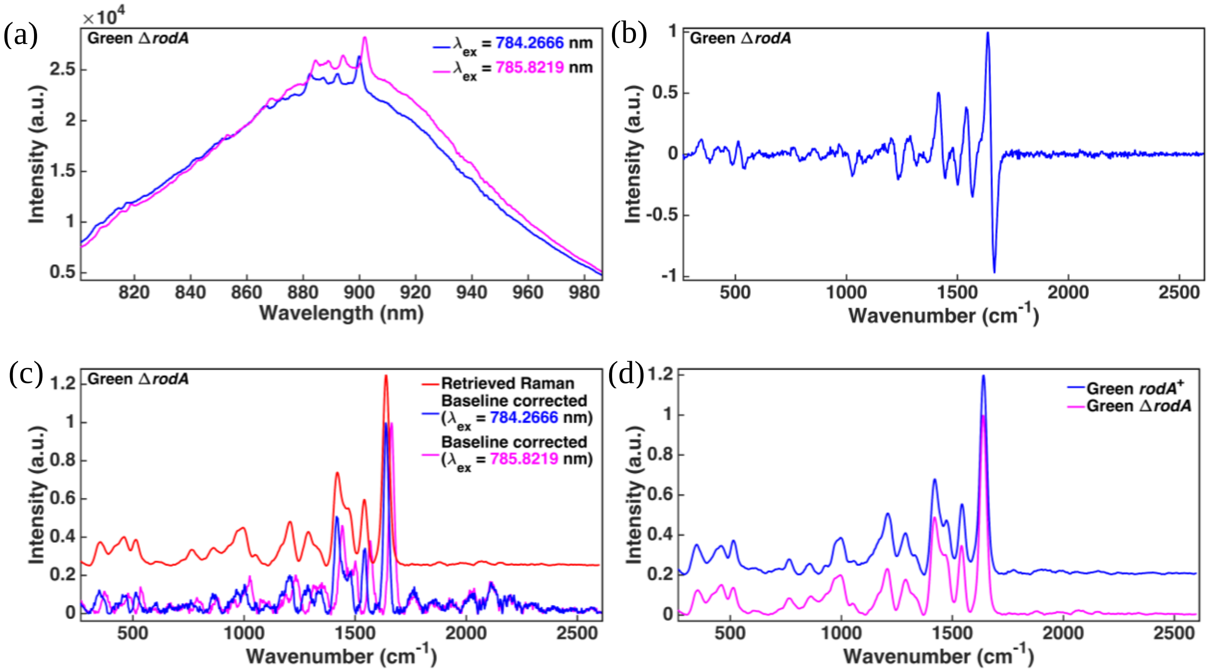

Figure S6. Results of a green Δ*rodA* conidium. (a) Raw spectra with two different excitation frequencies. The shift was ~25.2 cm^{-1}. (b) The difference spectrum between two normalized curves in (a). (c) Recovered Raman spectrum as compared with baseline-corrected data. (d) Comparison between the retrieved Raman spectra of a green $rodA^{+}$ conidium and its Δ*rodA* counterpart.

Figure S6(a), S6(b) and S6(c) show the results of a green Δ*rodA* spore. Visual comparison of the reconstructed Raman spectra of green $rodA^{+}$ and green Δ*rodA* conidia in Figure S6(d) indicates that they are virtually indistinguishable.

Appendix 9: Yellow Δ*rodA* conidia

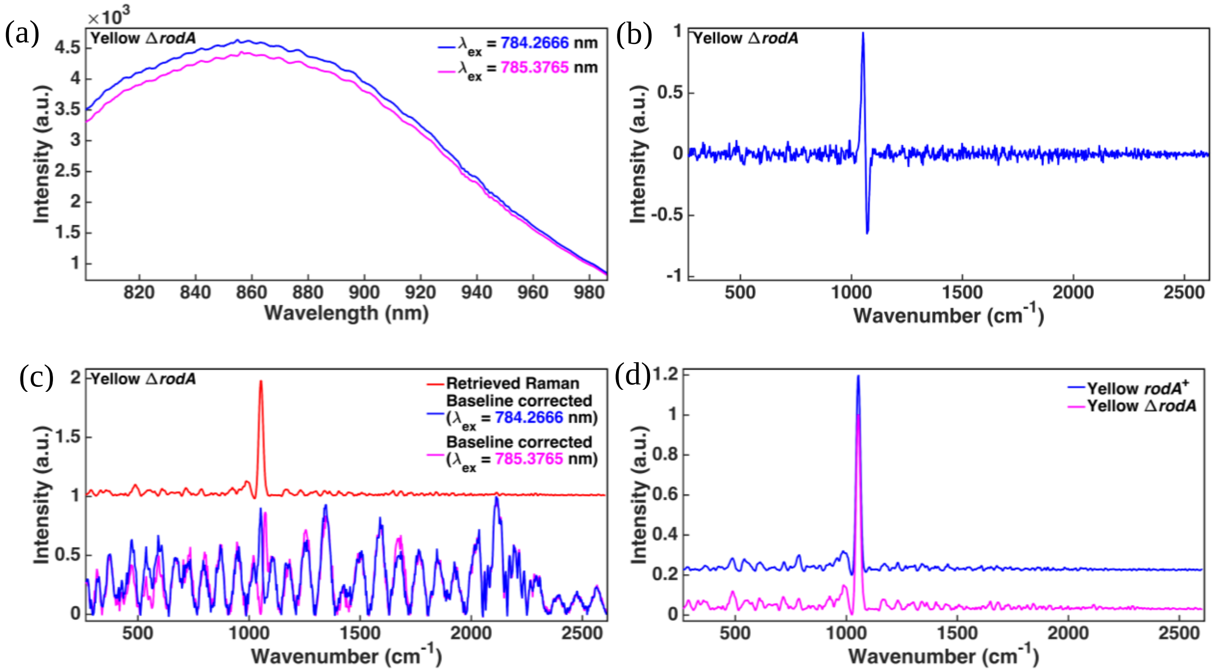

Figure S7. Results of the average of 100 yellow Δ*rodA* conidia. (a) Raw spectra with two different excitation frequencies. The shift was ~18.0 cm^{-1}. (b) The difference spectrum between two normalized curves in (a). (c) Retrieved Raman spectrum as compared with baseline-corrected data. (d) Comparison between the recovered Raman spectra of yellow *rodA*$^{+}$ and Δ*rodA* conidia.

The analysis of yellow Δ*rodA* spores in Figure S7 shows similar results to yellow *rodA*$^{+}$ spores. Visual comparison of the reconstructed Raman spectra in Figure S7(d) indicates that they are virtually indistinguishable.

Appendix 10: White Δ*rodA* conidia

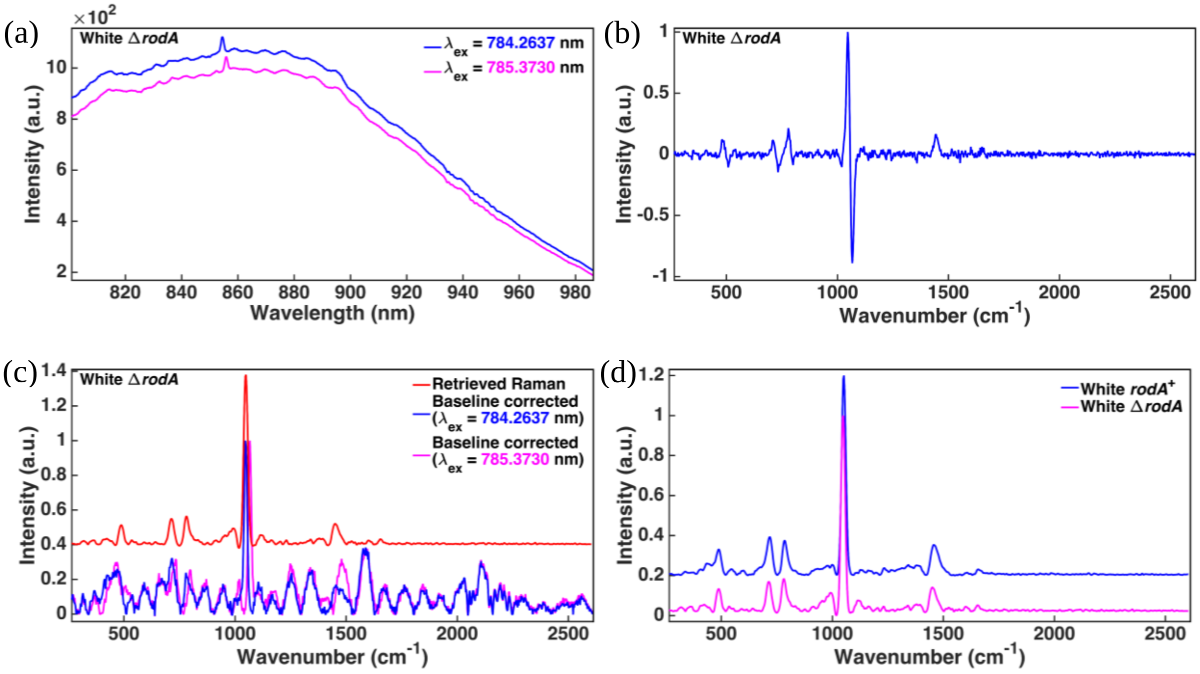

Figure S8. Results of the average of 100 white Δ*rodA* conidia. (a) Raw spectra with two different excitation frequencies. The shift was ~18.0 cm^{-1}. (b) The difference spectrum between two normalized spectra in (a). (c) Retrieved Raman spectrum as compared to baseline-corrected data. (d) Comparison between the reconstructed Raman spectra of white *rodA*$^+$ and Δ*rodA* conidia.

The analysis of white Δ*rodA* conidia in Figure S8 shows results similar to *rodA*$^+$ spores. Figure S8(d) illustrates that a visual comparison between the reconstructed Raman spectra of white *rodA*$^+$ and Δ*rodA* spores indicates they are virtually indistinguishable.

Appendix 11: Retrieved fluorescence of conidia

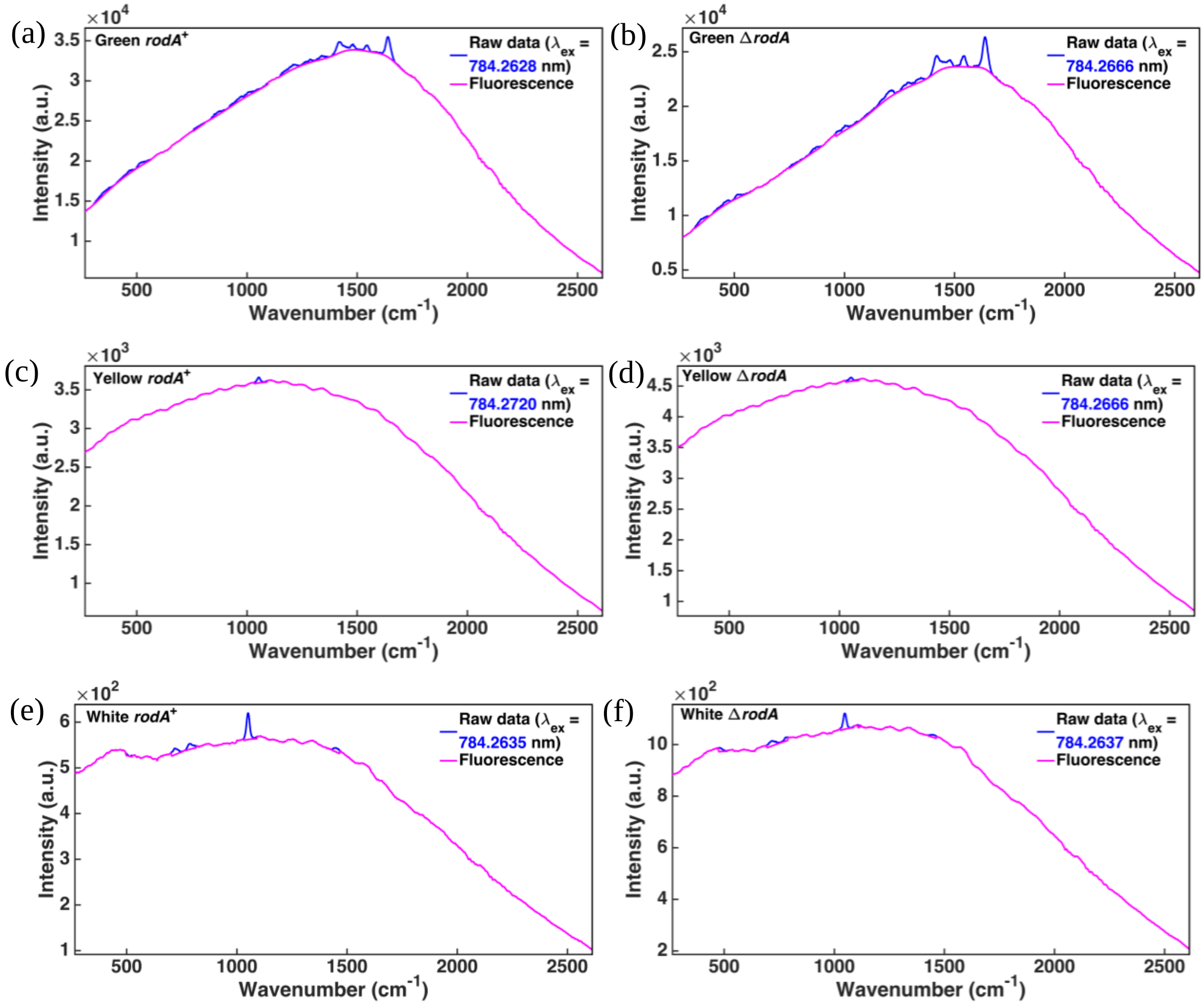

Figure S9. Comparison of raw data and reconstructed fluorescence spectra for (a) Green *rodA*$^+$, (b) Green Δ*rodA*, (c) Yellow *rodA*$^+$, (d) Yellow Δ*rodA*, (e) White *rodA*$^+$, (f) White Δ*rodA* conidia.

Figure S9 shows comparison of measured raw data and corresponding recovered fluorescence spectra for each strain.

Appendix 12: Optical images of *A. nidulans* conidia

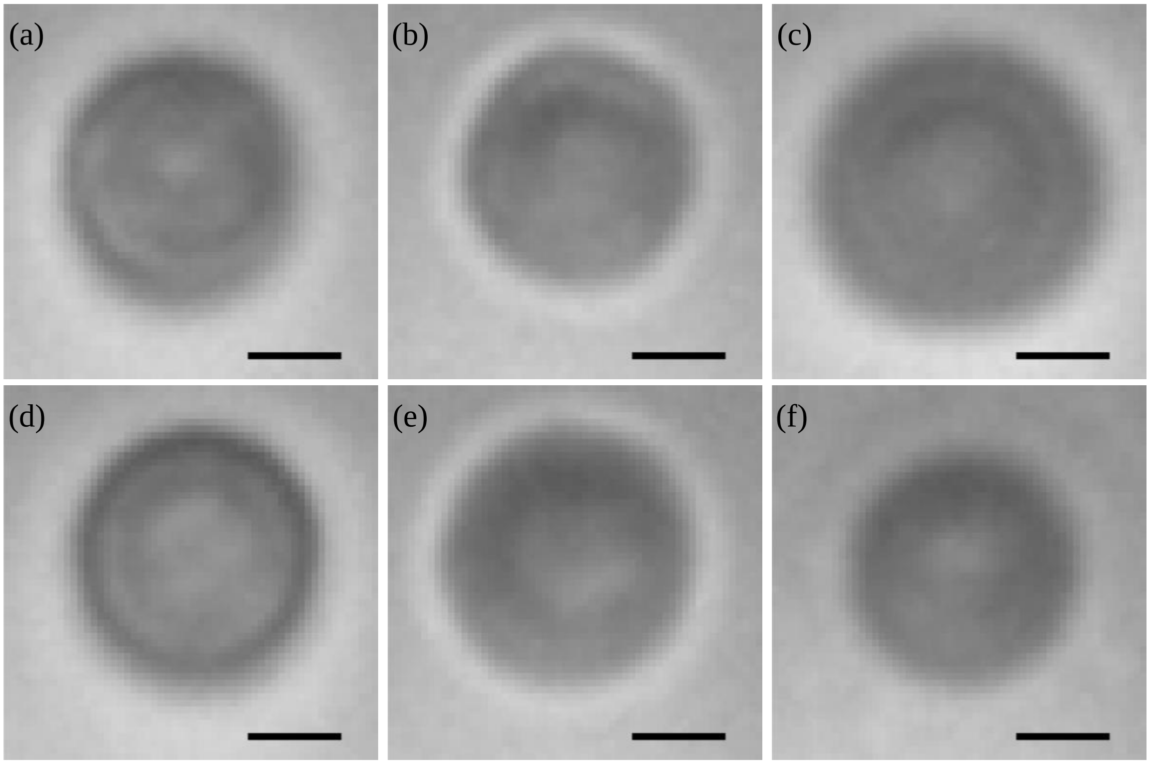

Figure S10. Optical images of a single green (a), yellow (b), and white (c) *rodA*$^+$ conidium of *A. nidulans* and corresponding green (d), yellow (e) and white (f) Δ*rodA* counterparts. Scale bar: 1.5 µm.

Figure S10 displays optical images of the three possible colors of conidia in *A. nidulans*. *rodA*$^+$ are in the top row (a-c), while the corresponding Δ*rodA* counterparts are in the bottom row (d-f). All images were token by the same objective (HCX PL Fluotar, 100X, N.A. 0.75, Leica) mentioned in the main text.

References

[1] Zhao, J., Carrabba M. M. & Allen, F. S. Automated fluorescence rejection using shifted excitation Raman difference spectroscopy. *Appl. Spectrosc.* **56**(7) 834-845 (2002).

[2] Klejnstrup, M.L., Frandsen, R.J.N., Holm, D.K., Nielsen, M.T., Mortensen, U.H., Larsen, T.O. & Nielsen, J.B. Genetics of polyketide metabolism in Aspergillus nidulans. *Metabolites* **2**(1) 100-133 (2012).

[3] Stringer, M.A., Dean, R.A., Sewall, T.C. & Timberlake, W.E. Rodletless, a new Aspergillus developmental mutant induced by directed gene inactivation. *Genes and Development* **5** 1161-1171 (1991).

[4] Grunbacher, A., Throm, T., Seidel, C., Gutt, B., Rohrig, J., Strunk, T., Vincze, P., Walheim, S., Schimmel, T., Wenzel, W. & Fischer, R. Six hydrophobins are involved in hydrophobin rodlet formation in Aspergillus nidulans and contribute to hydrophobicity of the spore surface. *PLOS ONE* **9**(4), e94546 (2014).

[5] Claverie-Martin, F., Diaz-Torres, M.R. & Geoghegan, M.J. Chemical composition and electron microscopy of the rodlet layer of Aspergillus nidulans conidia. *Curr. Microbiol.* **14,** 221-225 (1986).

[6] Zhao, L., Schaefer, D. & Marten, M.R.. Assessment of elasticity and topography of Aspergillus nidulans spores via atomic force microscopy. *Applied and Environmental Microbiology* **70**(2), 955-960 (2005).

[7] Wang, W., Major, A. & Paliwal, J. Grating-stabilized external cavity diode lasers for Raman spectroscopy—a review. *Appl. Spectrosc. Rev.* **47**(2), 116-143 (2012).

[8] Heim, P.J.S., Fan, Z.F., Cho, S.-H., Nam, K., Dagenais, M., Johnson, F.G., & Leavitt, R. Single-angled-facet laser diode for widely tunable external cavity semiconductor lasers with high spectral purity. *Electron. Lett.* **33**, 1387-1389 (1997).
